# Low-temperature synthesis of high-ordered anatase TiO_2_ nanotube array films coated with exposed {001} nanofacets

**DOI:** 10.1038/srep17773

**Published:** 2015-12-04

**Authors:** Jie Ding, Zhennan Huang, Jihao Zhu, Shengzhong Kou, Xiaobin Zhang, Hangsheng Yang

**Affiliations:** 1 State Key Laboratory of Silicon Materials, School of Materials Science and Engineering, Zhejiang University, Zheda Road 38, Hangzhou 310027, China; 2 State Key Laboratory of Advanced Processing and Recycling of Nonferrous Metals, Lanzhou University of Technology, Lanzhou 730050, China; 3 The Second Institute of Oceanography, State Oceanic Administration, Baochubei Road 36, Hangzhou 310012, China

## Abstract

High-ordered anatase TiO_2_ nanotube array films coated with exposed high-reactive {001} nanofacets were fabricated by a modified hydrothermal method using amorphous anodic TiO_2_ nanotube arrays (ATONAs) as starting materials. It was found that the reaction between gas phase HF and solid ATONAs played a key role in the transformation process from amorphous to anatase TiO_2_, and the TiO_2_ tubular structure kept unchanged during the surface modification with an exposed {001} facets up to 76.5%, which could be attributed to the low reaction temperature of 130 °C. Our study provided a novel route for the facile preparation of {001} facets exposed anatase TiO_2_.

1. Anatase TiO_2_ nanotube array films coated with exposed {001} nanofacets have been synthesized by a modified hydrothermal method.
2. Anatase TiO_2_ with 76.5% of exposed {001} facets was achieved at temperature as low as 130 °C.
3. A novel reaction route between HF gas and solid ATONAs played a key role.
4. The transformation depended on solid state atomic rearrangement.

Among the three main titanium dioxide (TiO_2_) polymorphs, anatase TiO_2_ has attracted much attention because of its unique electronic, optical and catalytic properties^1^,^2^,^3^, which showed various applications such as photocatalysis, photovoltaics, drug delivery, hydrogen production and lithium ion batteries^4^,^5^,^6^,^7^. Recently, the synthesis and application of anatase TiO_2_ with exposed {001} facets have been a hot topic^8^. Both theoretical and experimental studies revealed that the exposed (001) surfaces showed much higher chemical activities than the (101) surfaces in anatase TiO_2_ crystals^8^,^9^,^10^. Therefore, it is desirable to prepare TiO_2_ crystals with more reactive {001} facets exposed. Hydrothermal synthesis with F^−^ has been proven to be the most frequently used and the most effective method^8^,^9^,^10^,^11^,^12^,^13^. The main procedures for the exposal of {001} planes of anatase TiO_2_ included two steps: (i) the preparation of low surface energy {001} planes by terminating the dangling bonds with F^−^, at this stage, the photocatalytic activity of the TiO_2_ is still low; (ii) the removal of surface F^−^ by 600 °C annealing which produced F^−^ free exposed {001} planes. Note that the first step is essential for the exposal of {001} nanofacets. Up to now, a reaction temperature at least 200 °C is necessary for the hydrothermal preparation of anatase TiO_2_ with exposed {001} facets^8^,^9^,^10^,^11^,^12^,^13^.

Herein, we found that by using a modified hydrothermal method, which avoided the direct contact of ATONAs with HF solution^13^,^14^,^15^, thus the reaction between gas phase HF (but not acid HF solution) and solid ATONAs played a key role in the transformation process from amorphous to anatase TiO_2_, and TiO_2_ nanotube array films with exposed {001} facets up to 76.5% was successfully prepared at reaction temperature as low as 130 °C. After the surface F^−^ ions were removed by 2 h of 600 °C annealing^8^,^13^,^16^, the obtained F^−^ free TiO_2_ films with exposed {001} nanofacets showed much better photocatalytic activities than the original ATONAs for methyl orange (MO) degradation.

## Results

**Figure 1:**
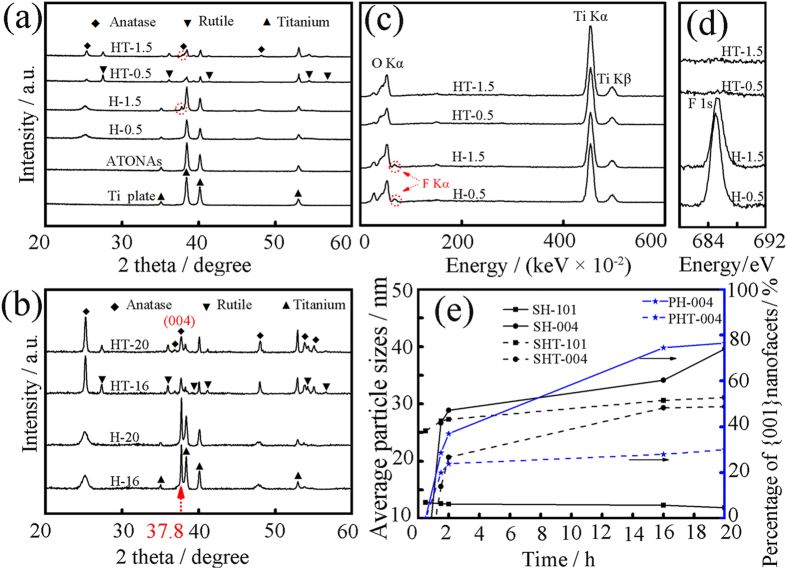
(**a**) and (**b**) XRD patterns of the samples before and after post annealing. The red dot circle is the location of (004) planes of the anatase TiO_2_. (**c**) EDS patterns of sample before (H-0.5 and H-1.5) and after (HT-0.5 and HT-1.5) post annealing, the red dot circle is the position of F Kα. (**d**) XPS spectra of two samples before and after post annealing, which clearly showed that the surface F^–^ ions were removed by annealing. (**e**) Structural information of the synthesized samples. SH-101 and SH-004: the average particle sizes calculated from the FWHM of (101) and (004) peak for samples before post annealing; SHT-101 and SHT-004: the average particle sizes calculated from the FWHM of (101) and (004) peak for samples after annealing; PH-004 and PHT-004: the percentage of exposed {001} nanofacets before and after annealing.

Figure 1a,b shows the XRD patterns of the as-synthesized samples with different preparation conditions. For ATONAs, only diffraction peaks of titanium were detected, indicating that the as grown ATONAs have amorphous structure^17^. After hydrothermal treatment, broad diffraction peaks at 25.3°, 37.8°, 48.2°, 53.9° and 55.2° were observed, which can be indexed to the (101), (004), (200), (105) and (211) reflections of anatase TiO_2_, and the diffraction peak intensities at 25.3° and 37.8° increased with preparation time, indicating the nucleation and growth of anatase TiO_2_ during 130 °C hydrothermal treatment (the XRD patterns of samples for 1.5 h and 2 h were shown in Fig. S1 in Supporting Information). Note that, the appearance of strong peak at 37.8°, which was attributed to (004) planes of anatase TiO_2_, suggested the presence of exposed {001} nanofacets^18^. Especially for H-16 and H-20 (Fig. 1b), the exposed {001} planes was estimated to be as high as 74.5% and 76.5%, respectively^19^. The EDS spectra in Fig. 1c indicated that the surface F^−^ ions were completely removed by 600 °C annealing^8^,^13^,^16^. XPS measurement also confirmed the successful removal of the surface F^−^ species, as shown in Fig. 1d. At the same time, diffraction peaks attributed to {101} planes sharpened, while the (004) peak at 37.8° weakened slightly, indicating that the loss of terminated F^−^ preferred the exposal of {101} planes. The average grain size estimated from FWHM of diffraction peaks at 25.3° and 37.8° was 12.2 nm and 34.1 nm for H-16, and 30.6 nm and 29.3 nm for HT-16, respectively as shown in Fig. 1e. By the way, the post annealing also induced the formation of rutile nanoparticles, as a result, diffraction peaks of 27.5°, 36.1°, 39.3° and 54.3° appeared^20^,^21^.

**Figure 2:**
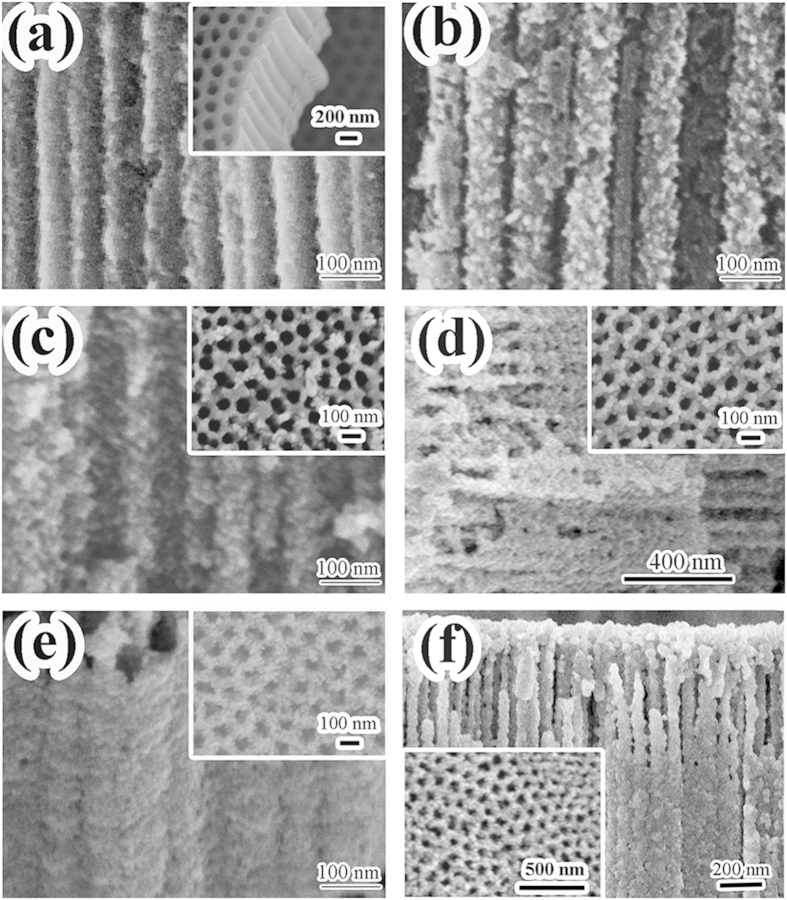
Typical SEM images of the samples: (a) H-0.5, (b) HT-0.5, (c) H-16, (d) HT-16, (e) H-20, (f) HT-20. The inset in (a) is as-prepared ATONAs, and other insets are morphology of film surface. And their low-magnification images are represented in ESI.

Figure 2 shows SEM images of samples prepared under different conditions. The inset of Fig. 2a is SEM image showing the morphology of the as-prepared ATONAs, which were highly ordered and were grown vertically to the titanium substrate. Figure 2a is the ATONAs after 0.5 h hydrothermal treatment, the tubular structure was maintained with small particles coated on the surface (~8 nm estimated from the FWHM of peak at 25.3°). Even the hydrothermal treatment time increased to 20 h, the tubular structure still kept unchanged as the cross section image shown in Fig. 2e. Amazingly, after 600 °C annealing, the surface coated nanoparticles grew up to be nanofacet like grains (insets of Fig. 2d,f ), and the tubular structure was still maintained as shown in Fig. 2b,d,f, which provided an extremely large surface area compared to the normal TiO_2_ films (low-magnification SEM images were shown in Figs. S2 to S6).

**Figure 3:**
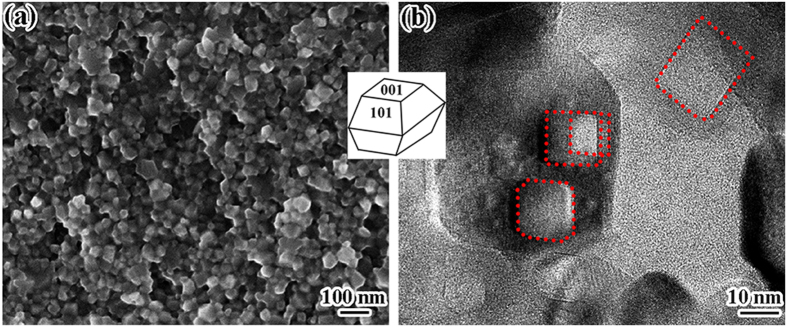
(**a**) SEM and (**b**) TEM image of HT-2. The inset is a model of equilibrium shape of anatase TiO_2_ with exposed {101} and {001}.

Figure 3a shows SEM image of nanoparticles with truncated bipyramidal or trapezoidal shapes and even some with cubic morphology of anatase TiO_2_ coated on the surface of HT-2, which could be attributed to different degree of truncated octahedral. Figure 3b shows typical TEM image of some nanoparticles, which have regular facet-like geometry with exposed flat square^22^. All SEM and TEM images indicated the successful exposal of F^−^ free (004) planes after annealing^8^. Figure 1e summarized the grain size and the evaluation of percentage of exposed {001} planes, according to early estimation method^19^,^23^,^24^, TiO_2_ with exposed {001} facets up to 76.5% was successfully prepared.

**Figure 4:**
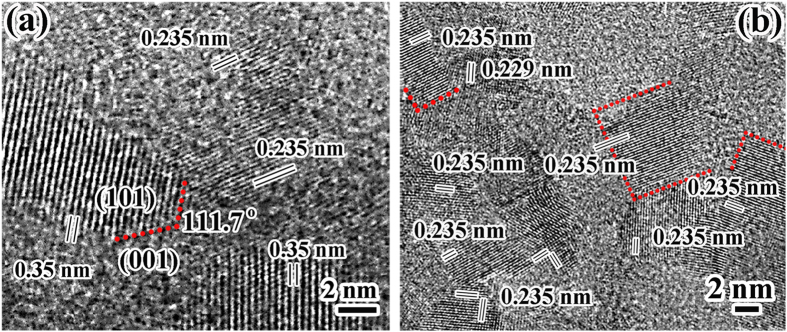
HRTEM images of (a) H-2 and (b) HT-2. The 0.235 nm and 0.35 nm are the distances between (004) planes and (101) plane of anatse TiO_2_, respectively. The 111.7° is the angle between (004) plane and (101) plane of anatase TiO_2_.

Figure 4a shows the HRTEM image of H-2 sample, nanoparticles with grain size in the orders of several nanometers could be observed, the crystal lattice with a spacing of 0.235 nm (004), and 0.35 nm (101) confirmed the anatase TiO_2_ nanoparticles^24^. Moreover, the observed (004) planes are parallel to the particle surface, which presented direct evidence for the existence of {001} exposed nanofacets. Evidently, the amorphous ATONAs partially transformed into anatase TiO_2_ with exposed {001} nanofacets by hydrothermal treatment at 130 °C. Figure 4b is a typical HRTEM image of HT-2, in this selected observation window, almost all grains had spacing of 0.235 nm (parallel to the grain surface); indicated the high percentage of exposed {001} planes. By the way, a rutile TiO_2_ grain with a spacing of 0.229 nm belonging to (200) planes was also detected, consistent with XRD results shown in Fig. 1. HRTEM images of HT-20 were shown in Fig. S7.

## Discussion

Normally, anatase TiO_2_ grains are dominated by {101} facets rather than {001} facets, the latter is more active but with high surface energy^25^,^26^,^27^,^28^,^29^. When the surface was terminated with F^−^, the expose of {001} is energetically preferable to {101}, and this was proven theoretically and experimentally by using hydrothermal method^8^,^9^,^10^, by which the Ti precursors were directly immersed into F^−^/HF containing solutions^30^. A dissolution-precipitation or dissolution-recrystallization process was proposed to describe the transformation from {101} to {001} facets^31^,^32^,^33^. Here we modified the hydrothermal method by separating the ATONAs from the acid HF solution^30^, accordingly, the reaction between gas phase HF and solid ATONAs becomes the only route for the transformation from amorphous to anatase TiO_2_. From the XRD spectra shown in Fig. 1, a low temperature of 130 °C was enough to induce this transformation. This could be attributed to the use of amorphous TiO_2_ as starting materials, which reduced the activation energy for atom rearrangement. The tubular structure of ATONAs was kept unchanged after hydrothermal treatment, which provided additional evidence that this transformation could not be explained through dissolution-precipitation or dissolution-recrystallization process, which destroyed the initial tubular structure^31^,^32^,^33^. The driving force for this transformation could be the surface energy change caused by F^−^ termination, and {001} nanofacets were preferred finally^8^,^9^,^10^,^11^,^12^,^13^,^14^. From literature, the solid state transformation via atomic rearrangement in amorphous matrix is frequently proposed^34^,^35^. And the synthesis of anatase TiO_2_ with exposed {001} facets by heat treatment (450 °C) using amorphous ATONAs as starting materials was achieved^13^,^16^. Here we demonstrated that a solid-state phase transformation from TiO_2_ {101} to {001} facets could be achieved at a low temperature of 130 °C.

**Figure 5:**
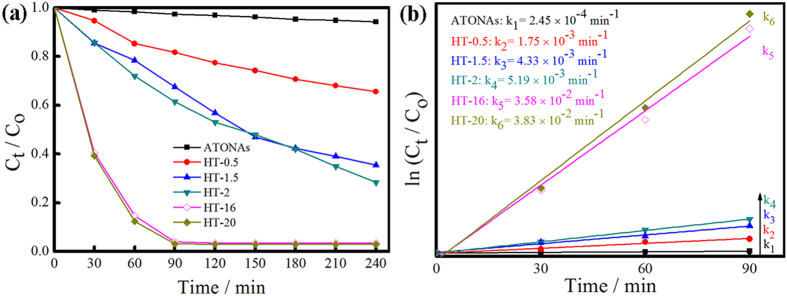
Photocatalytic activities of ATONAs, HT-0.5, HT-1.5, HT-2, HT-16, and HT-20 (a), and their corresponding pseudo-first-order kinetics (b).

After the surface F^−^ ions were removed by 600 °C post annealing, F^−^ free {001} facets with high reactivity were achieved. Figure 5a shows the photocatalytic activities of ATONAs, HT-0.5, HT-1.5, HT-16, and HT-20, respectively. The corresponding degradation efficiency increased with the percentage of {001} nanofacets, and more than 97% of MO could be removed in 90 min over HT-16 and HT-20. According to Langmuir-Hinshelwood model^36^, the reaction rate coefficient k of HT-20 (k_6_ = 3.83 × 10^−2^ min^−1^) is 150 times higher than that of ATONAs (k_1_ = 2.45 × 10^−4^ min^−1^) (Fig. 5b), which demonstrated the excellent photocatalytic activities of TiO_2_ nanotube array films with exposed {001} nanofacets obtained in this study, coincided well with earlier reports^13^,^15^.

## Conclusions

In summary, anatase TiO_2_ nanotube array films with exposed {001} nanofacets were successfully prepared by a modified low-temperature hydrothermal method at 130 °C. The novel reaction route between gas phase HF and solid ATONAs was demonstrated which played a key role for the solid state transformation process from amorphous to anatase TiO_2_, after the surface dangling bonds were terminated with F^−^ ions, {001} facets became energetically favored. F^−^ free {001} facets with high reactivity could be achieved via 600 °C post annealing. The prepared anatase TiO_2_ nanotube array films with exposed {001} nanofacets exhibited enhanced photocatalytic activity for methyl orange (MO) degradation under ultraviolet light (UV), which could be attributed to the improvement of charge separation derived from the synergy effect between {001} and {101} facets.

## Methods

### Preparation of well-aligned anodic TiO_2_ nanotubes (ATONAs)

All reagents (Sinopharm, analytical grade, Shanghai, China) were used without further purification. Well-aligned ATONAs on a Ti substrate were fabricated via traditional electrochemical anodization^37^. Briefly, the anodic growth was conducted using a home-made two electrode electrochemical cell under a constant voltage of 40 V at room temperature, titanium plates (Ti, 90 mm × 40 mm × 0.2 mm, 99.9% purity, Baoji, Shanxi, China) were used as working electrode and a graphite plate as the counter electrode, the electrolyte was composed of 3 wt% NH_4_F and 0.5 v% H_2_O dissolved in the ethylene glycol. The obtained well-aligned ATONAs were rinsed with ethylene glycol and ethanol to remove the residual electrolyte solution followed by drying at 80 °C for later use (inset of Fig. 2a).

### Exposing of {001} nanofacets

The well-aligned ATONAs were treated at 130 °C in an autoclave. In a typical experiment, 6 ml of hydrofluoric acid solution with pH = 3 was transferred to a Teflon-lined autoclave (capacity: 25 mL), then the as-synthesized ATONAs, which were cut into 2 cm × 2 cm dices, were fixed by Teflon holders 1 cm above the solution to avoid dipping the ATONAs into the solution. Then, the autoclave was sealed and heated at 130 °C for 0.5 h to 20 h. After the autoclave was cooled down to room temperature, the products were washed with ethylene glycol, ethanol and deionized water, and then dried in an oven at 80 °C for 0.5 h, the samples were labeled as H-t (where t stood for the hydrothermal time). Then, samples were heated at 600 °C for 2 h in air with a heating rate of 2°/min to obtain {001} nanofacets exposed films, and the final samples were labeled as HT-t.

### Characterization

X-ray diffraction (XRD) patterns were recorded on a Philips XD-98 X-ray diffractometer with Cu Kα radiation (λ = 0.15406 nm). The morphology of the samples was characterized by A ZEISS ULTRA 55 scanning electron microscopy (SEM) equipped with energy dispersive spectrometer (EDS) and transmission microscopy (TEM, FEI Tecnai G^2^ F20 S-TWIN, FEI Inc., America). X-ray photoelectron spectroscopy (XPS) data were obtained using a Thermo ESCALAB 250Xi (Thermo Fisher Scientific), the X-ray source was an Al Kα radiation, and all binding energies were referenced to the 284.8 eV C1s.

### Photocatalytic activity

The photocatalytic activities were evaluated by degrading the methyl orange (MO, 0.15 mg/L) as the model organic pollutants in aqueous solutions, three plates of {001} exposed TiO_2_ films (size: 2 cm × 2 cm, TiO_2_ weight: ca. 1.8 mg) were dipped in 30 mL of MO solution and then were irradiated by a 125 W mercury lamp which irradiated the light of 365 nm. Before irradiation, the system was put in a darkroom for 0.5 h with magnetic stirring to ensure adsorption and desorption equilibrium between the samples and organic molecules. The photodegradation experiments were performed in an open quartz vessel under continuously stirring. During degradation, 2 mL of MO were taken every 30 min and the concentration of MO was measured by UV-3600 spectrophotometer (Shimadzu, Japan) at 463 nm^38^. The influence of sampling was compensated in degradation efficiency calculation.

## Additional Information

**How to cite this article**: Ding, J. *et al.* Low-temperature synthesis of high-ordered anatase TiO_2_ nanotube array films coated with exposed {001} nanofacets. *Sci. Rep.* **5**, 17773; doi: 10.1038/srep17773 (2015).

## Supplementary Material

Supporting Information
