# Supplementary material for: Low-temperature synthesis of high-ordered anatase TiO2 nanotube array films coated with exposed {001} nanofacets
Source: Sci Rep. 2015 Dec 4;5:17773. doi: 10.1038/srep17773 (PMC4669522; doi:10.1038/srep17773)
Supplement: Supporting Information [file srep17773-s1.pdf]

Supporting Information

Low-temperature synthesis of high-ordered anatase TiO_2 nanotube array films coated with exposed {001} nanofacets

Jie Ding [1], Zhennan Huang[1, 2], Jihao Zhu[3], Shengzhong Kou[2], Xiaobin Zhang[1], Hangsheng Yang[1*]

[1] State Key Laboratory of Silicon Materials, School of Materials Science and Engineering, Zhejiang University, Zheda Road 38, Hangzhou 310027, China

[2] State Key Laboratory of Advanced Processing and Recycling of Nonferrous Metals, Lanzhou University of Technology, Lanzhou 730050, China

[3] The Second Institute of Oceanography, State Oceanic Administration, Baochubei Road 36, Hangzhou 310012, China

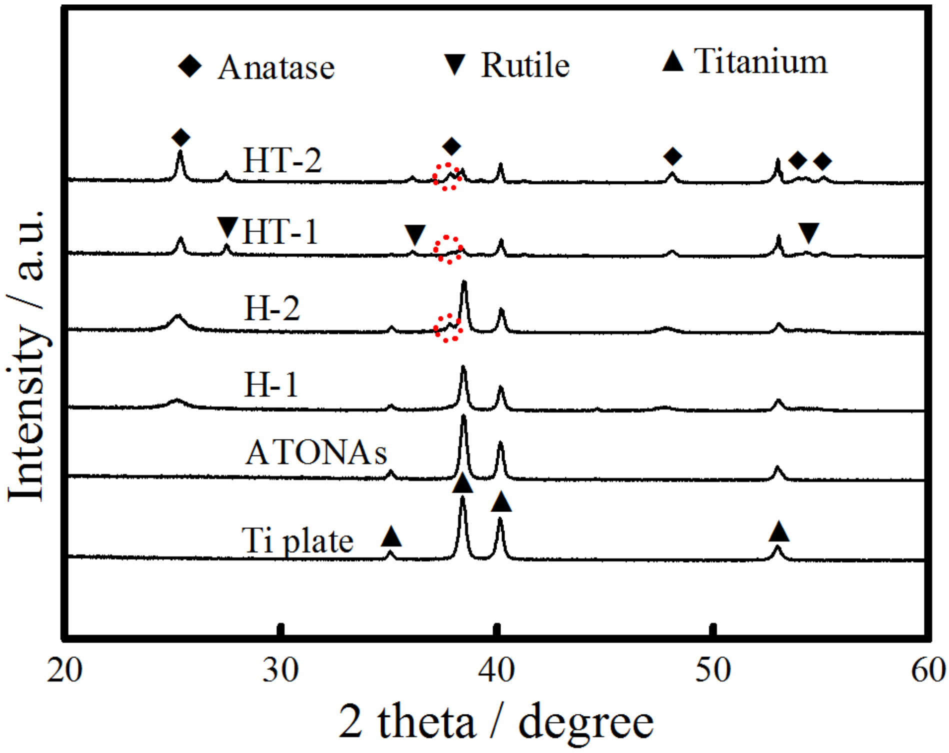

Figure S1. The XRD patterns of Ti plate, ATONAs, H-1, H-2, HT-1 and HT-2. The red dot circles indicate the existence of exposed (001) nanofacets of anatase TiO_2.

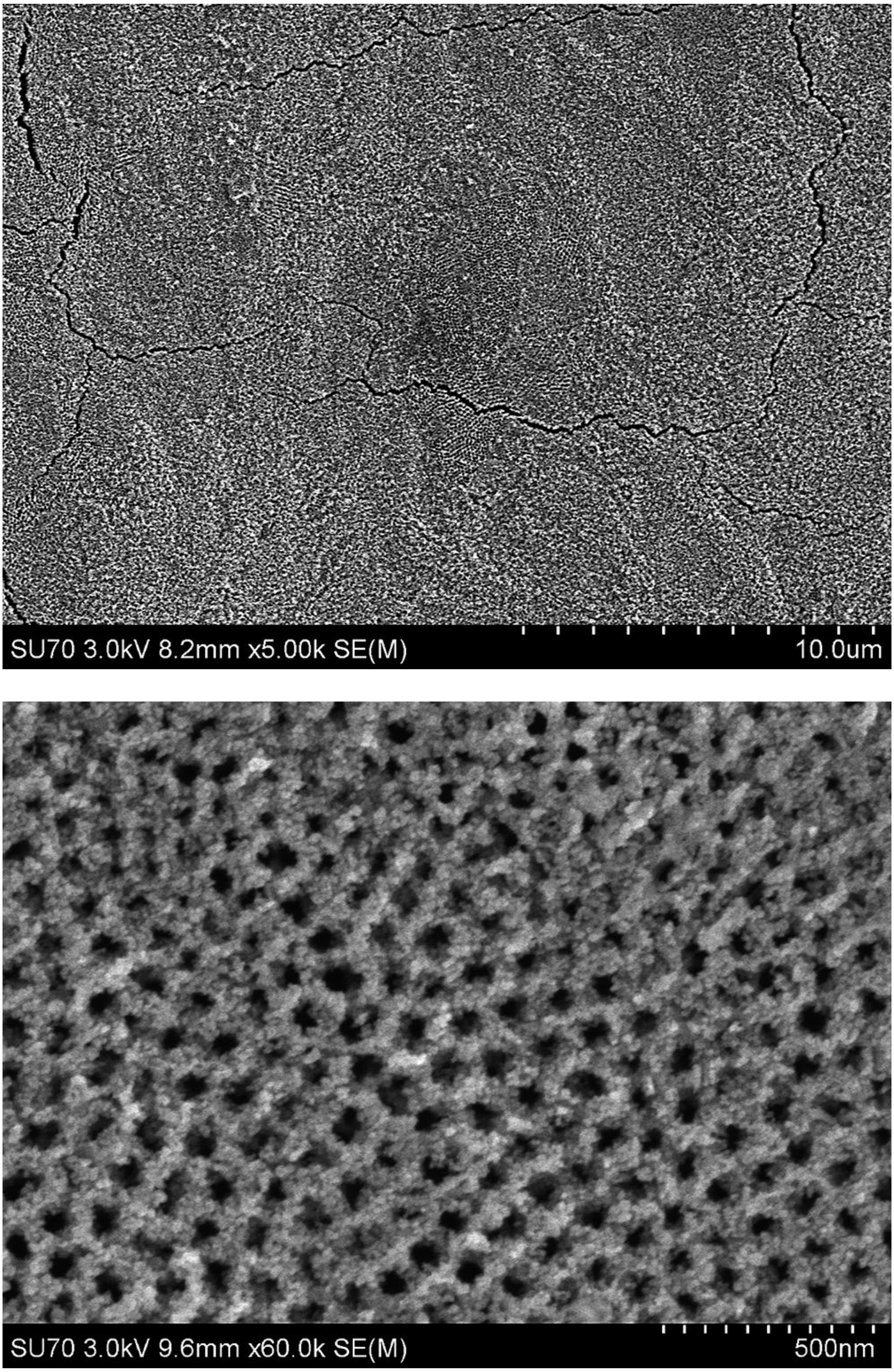

Figure S2. Top view of the as-synthesized H-16 films coated with {001} nanofacets.

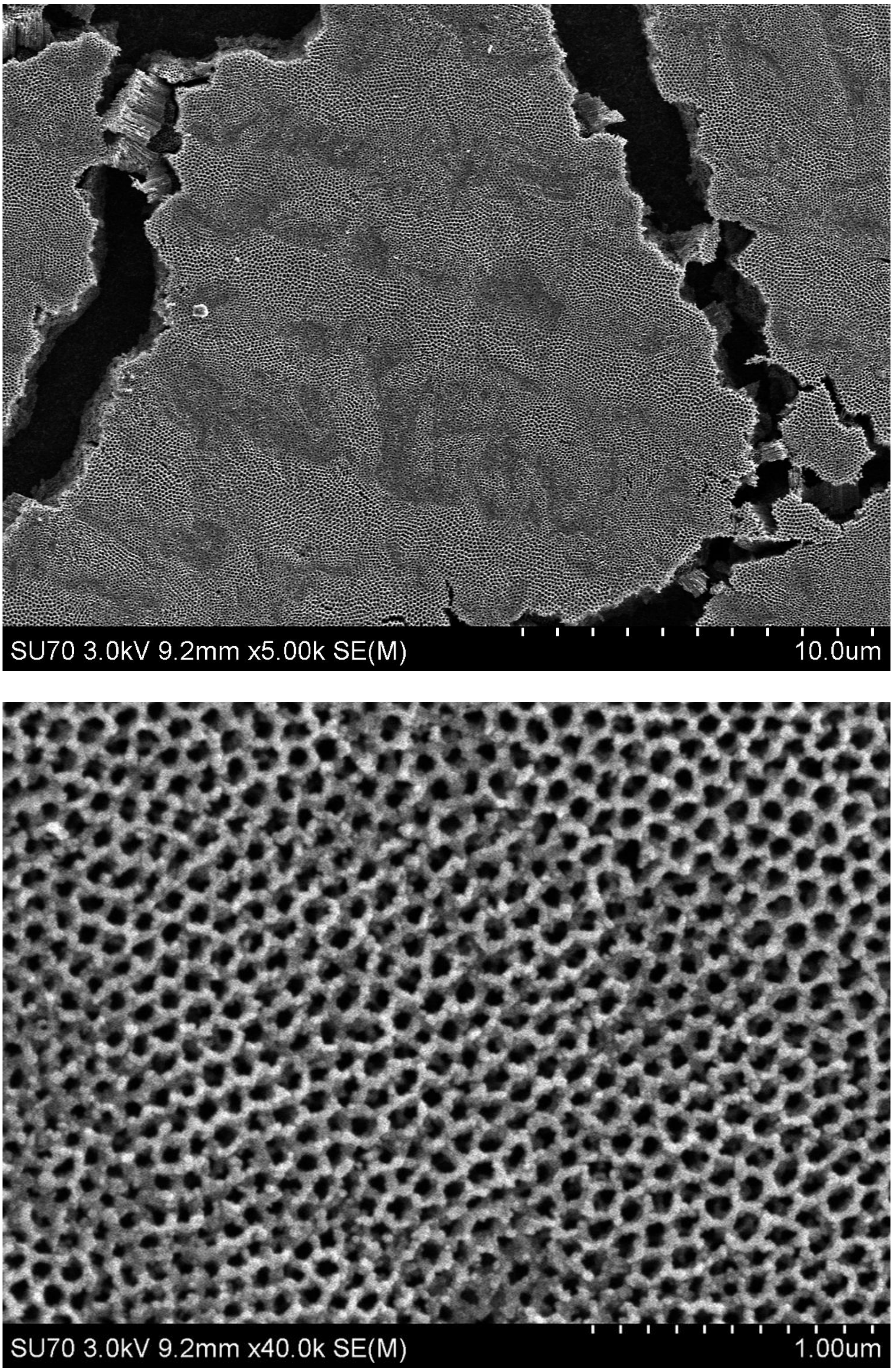

Figure S3. Top view of the as-synthesized HT-16 films coated with {001} nanofacets. After 600°C post annealing, the facet-like particles became visible, while the tubular structure kept unchanged with some small cracks.

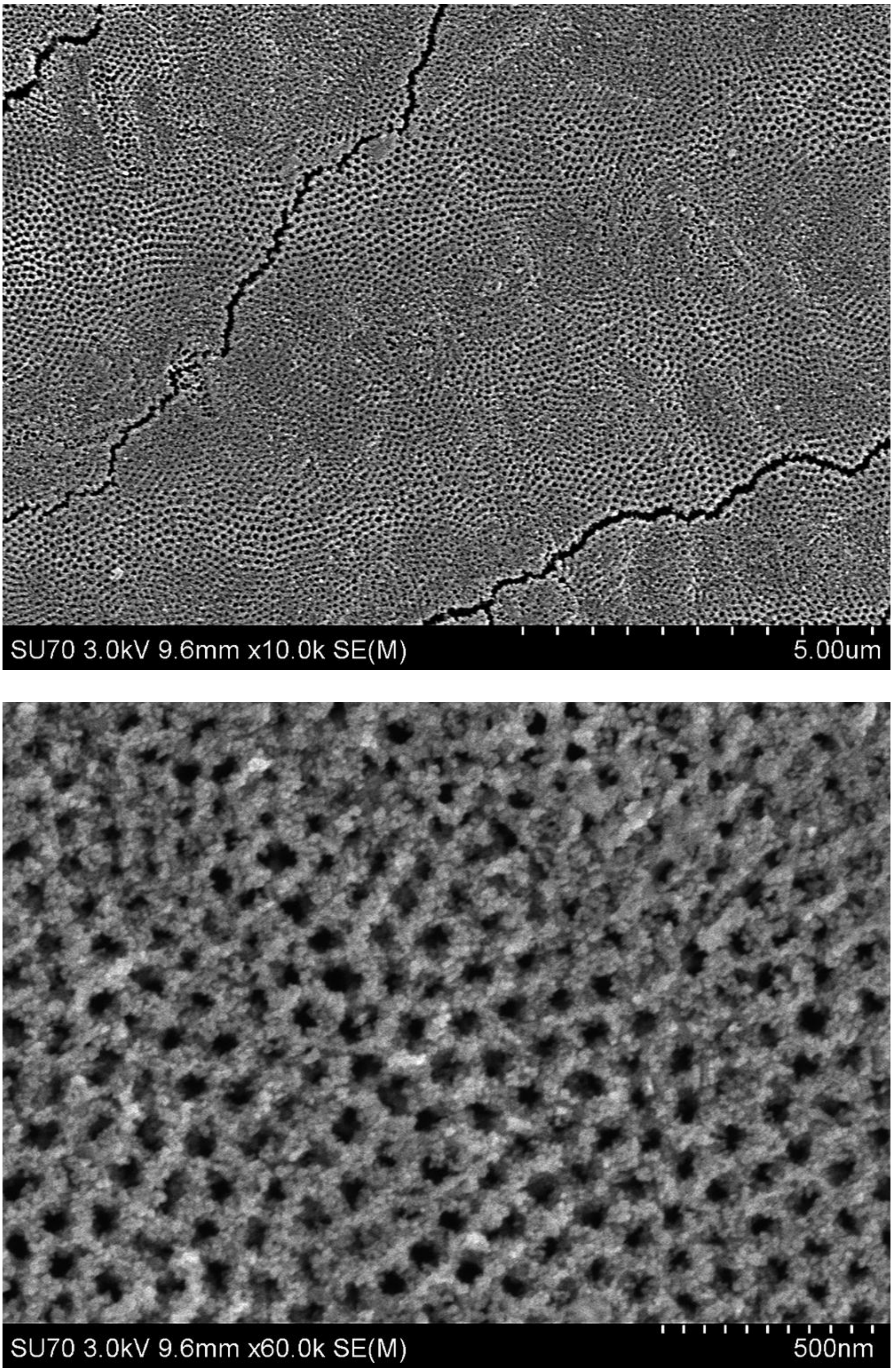

Figure S4. Top view of the as-synthesized H-20 films coated with {001} nanofacets.

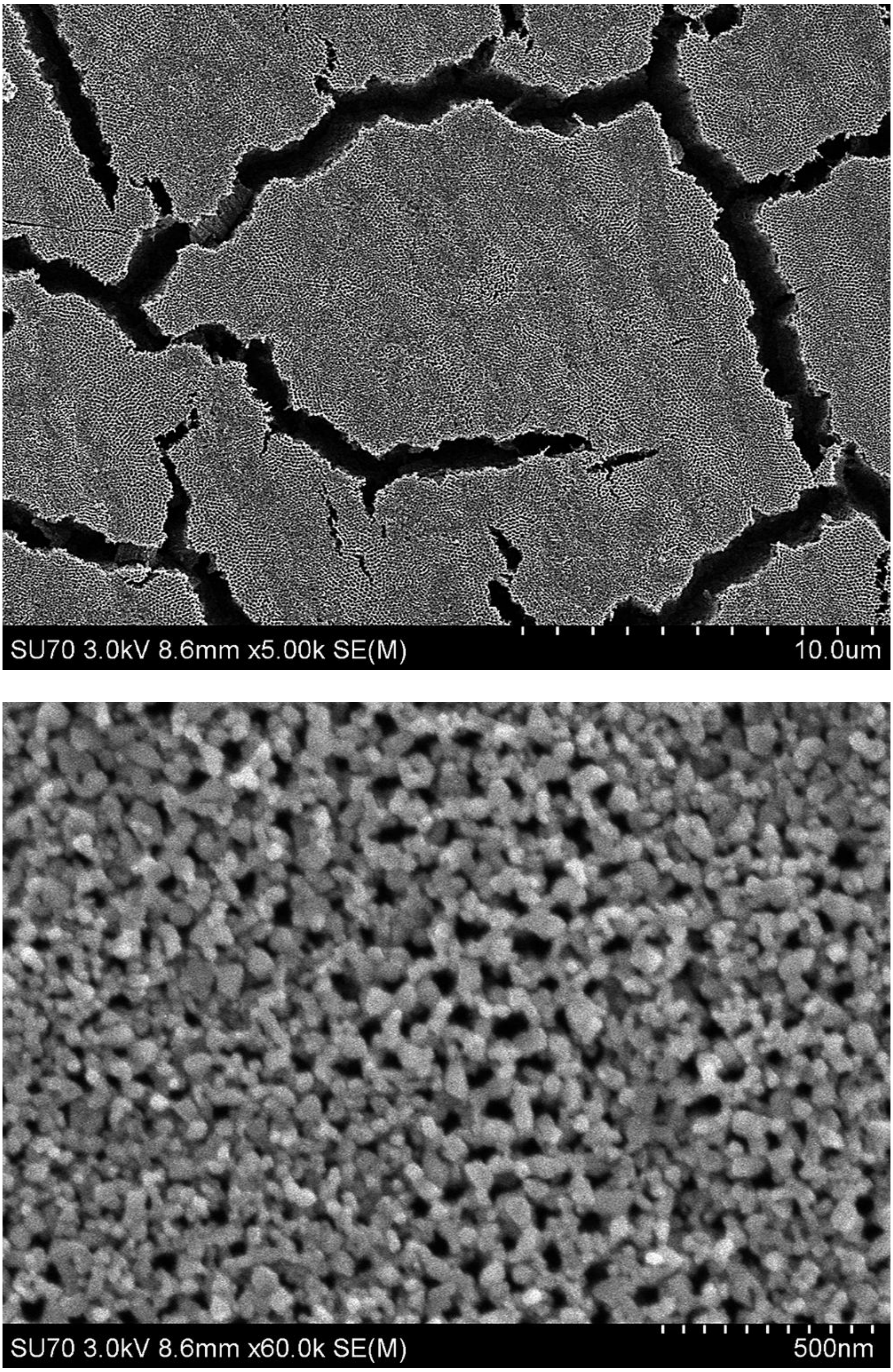

Figure S5. Top view of the as-synthesized HT-20 films coated with {001} nanofacets. Similarly, the tubular structure kept unchanged with small cracks.

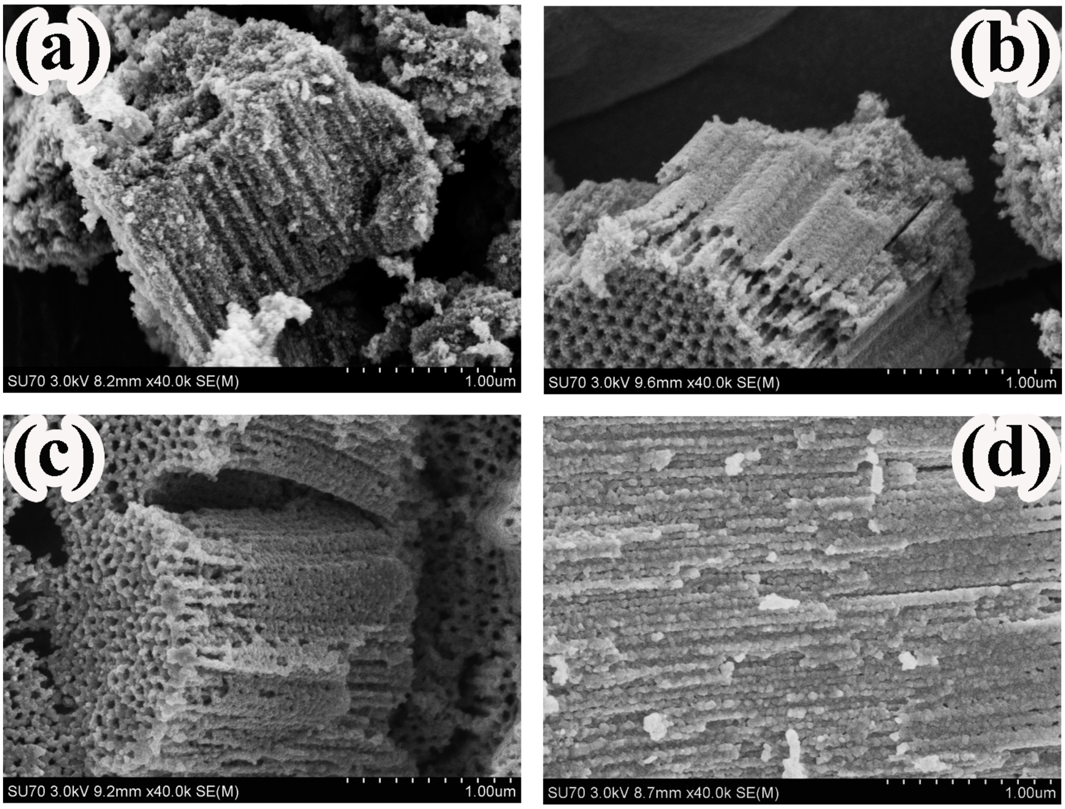

Figure S6. Side view of the as-synthesized films coated with {001} nanofacets. (a) H-16, (b) H-20, (c) HT-16, and (d) HT-20.

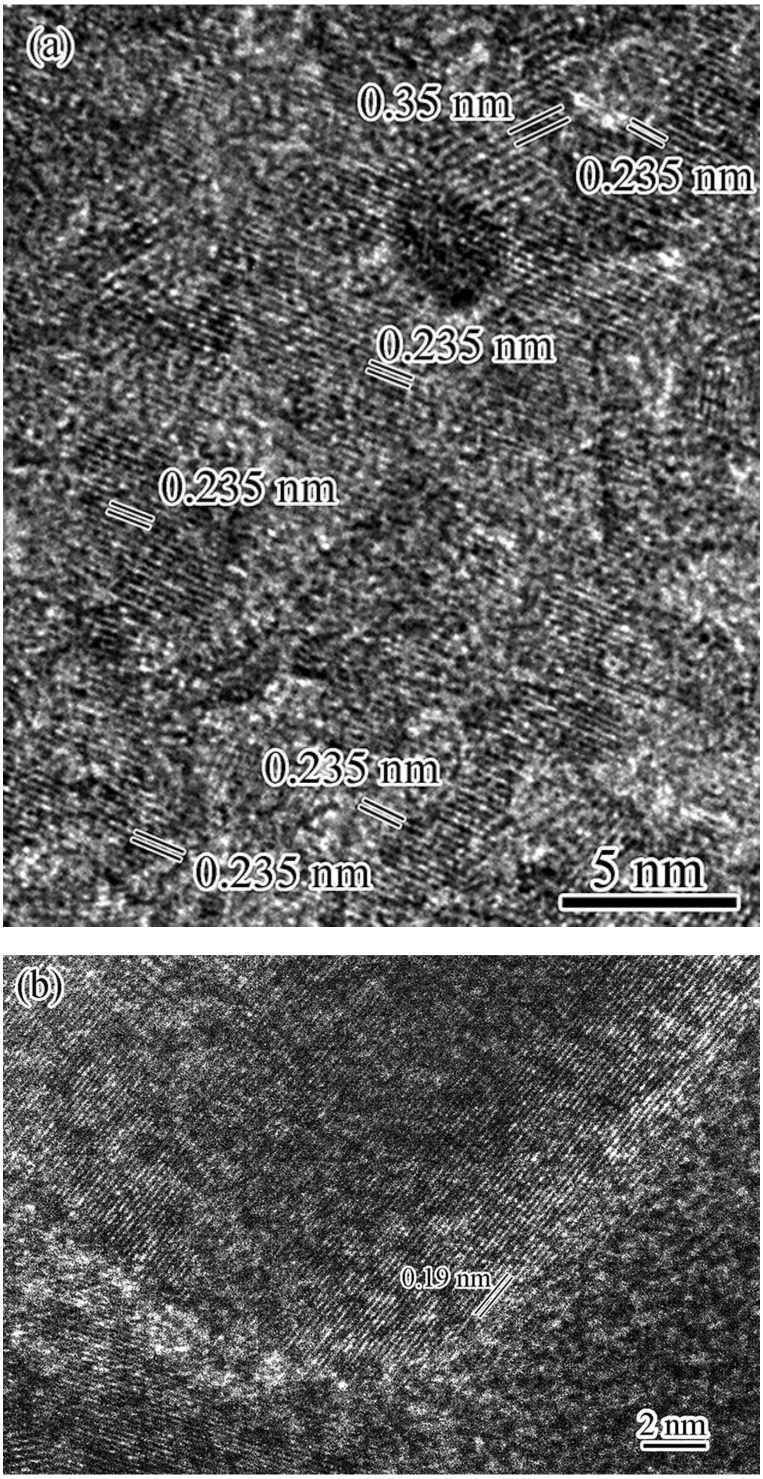

Figure S7. HRTEM images of HT-20. (a) d-spacing of 0.235nm attributed to (001) was frequently observed in this observation window; (b) an isolated particle with a d-spacing of 0.19 nm attributed to (200) could be observed.
